# Pre-pregnancy body mass index and risk of macrosomia: glycemic status-specific thresholds and subgroup interactions in a prospective cohort

**DOI:** 10.3389/fnut.2025.1633088

**Published:** 2025-06-27

**Authors:** Yuhang Wu, Hanyu Xiao, Lizhang Chen, Jiabi Qin, Tingting Wang

**Affiliations:** ^1^Department of Epidemiology and Health Statistics, Xiangya School of Public Health, Central South University, Changsha, Hunan, China; ^2^School of Dentistry, Stomatological Hospital, Tianjin Medical University, Tianjin, China; ^3^Hunan Provincial Key Laboratory of Clinical Epidemiology, Xiangya School of Public Health, Central South University, Changsha, Hunan, China; ^4^School of Public Health, Kunming Medical University, Kunming, China; ^5^NHC Key Laboratory of Birth Defect for Research and Prevention, Hunan Provincial Maternal and Child Health Care Hospital, Changsha, Hunan, China

**Keywords:** macrosomia, pre-pregnancy body mass index, gestational diabetes mellitus, prospective cohort, maternal metabolic health, risk stratification

## Abstract

**Background:**

Macrosomia, a critical perinatal complication, is closely linked to maternal obesity and gestational diabetes mellitus (GDM). However, the extent to which GDM status modifies the association between pre-pregnancy body mass index (BMI) and macrosomia, particularly across demographic subgroups, remains poorly understood. This study aimed to quantify glycemic status-specific risk thresholds and explore subgroup interactions in a large prospective cohort.

**Methods:**

In this prospective cohort study, 34,031 women initiating antenatal care before 14 weeks of gestation were enrolled at a tertiary hospital in Central China (2013–2019). Participants were stratified by GDM status and pre-pregnancy BMI categories. Multivariable logistic regression, restricted cubic spline (RCS) models, and interaction analyses evaluated associations between BMI and macrosomia (birth weight ≥ 4,000 g), adjusting for sociodemographic, behavioral, and clinical covariates.

**Results:**

Macrosomia incidence was markedly higher in GDM (6.2%) vs. non-GDM pregnancies (3.6%). Adjusted models revealed a steeper dose–response gradient in GDM: each 1-unit BMI increase conferred 24% higher odds (aOR: 1.24 [95% CI 1.20, 1.28]) in GDM vs. 13% (aOR: 1.13 [1.11, 1.15]) in non-GDM. Obesity amplified risk 6.80-fold (aOR: 6.80 [4.02, 11.51]) in GDM vs. 4.70-fold (aOR: 4.70 [3.12, 7.10]) in non-GDM. RCS models identified nonlinear trajectories in both GDM and non-GDM pregnancies (reference level: 22.94 kg/m^2^ for GDM and 25.10 kg/m^2^ for non-GDM). Significant interactions were observed in GDM pregnancies, and the association between pre-pregnancy BMI values and macrosomia was stronger in women < 35 years (aOR: 1.29 vs. ≥35 years, aOR: 1.15), primigravida (aOR: 1.61 vs. multigravida, aOR: 1.18), primiparous (aOR: 1.36 vs. multiparous, aOR: 1.18), and female infants (aOR: 1.29 vs. male, aOR: 1.20). In non-GDM pregnancies, only parity (primiparous, aOR: 1.08 vs. multiparous, aOR: 1.19) and gravidity (primigravida, aOR: 1.05 vs. multigravida, aOR: 1.19) modified the pre-pregnancy BMI-macrosomia relationship.

**Conclusion:**

GDM status modifies pre-pregnancy BMI-associated macrosomia risks, with distinct thresholds and subgroup vulnerabilities. These findings necessitate glycemic status-specific clinical guidelines and precision interventions targeting high-risk subgroups. Universal preconception weight optimization remains pivotal for non-GDM populations. This study underscores the urgency of integrating metabolic and demographic heterogeneity into perinatal care to mitigate the dual epidemics of overweight/obesity and GDM.

## 1 Introduction

Macrosomia, defined as a birth weight exceeding 4,000 g, presents significant clinical and public health challenges due to its association with adverse perinatal outcomes. Neonatal complications, including shoulder dystocia, birth trauma, and metabolic disturbances, are further exacerbated by long-term risks of childhood obesity and cardiometabolic disorders (1–4). Maternal risks associated with macrosomia include elevated likelihoods of cesarean delivery, postpartum hemorrhage, and pelvic floor injury (2, 4, 5). Additionally, a pilot evaluation estimate suggested that the short-term direct costs for neonatal complications of a macrosomic birth are $3,800 (6). Globally, macrosomia affects 5–20% of live births, with marked geographic and socioeconomic disparities (7). Especially in China, a super populous country, an increasing trend of macrosomia prevalence has been documented, from 6.9% in 2007 to 7.8% in 2017 (8). Given the increasingly immense societal and individual burden caused by macrosomia, there is an urgent need for preventive strategies targeting modifiable risk factors.

Pre-pregnancy body mass index (BMI) has emerged as a critical determinant of fetal growth. The dose–response relationship between pre-pregnancy BMI and macrosomia risk has been well-documented across BMI categories. A meta-analysis of 1.4 million pregnancies demonstrated that pre-pregnancy overweight (BMI 25.0–29.9 kg/m^2^) and obesity (BMI ≥ 30 kg/m^2^) were associated with 1.7-fold and 2.9-fold increased risks of macrosomia, respectively, compared to normal pre-pregnancy BMI (9). Conversely, pre-pregnancy underweight status (BMI < 18.5 kg/m^2^) was linked to a nearly 50% reduction in risk (8). The pathophysiological basis for this association is hypothesized to involve chronic maternal hyperglycemia and insulin resistance, exacerbated by excess adiposity (10). Adipose tissue-derived cytokines, such as leptin and TNF-*α*, may impair placental nutrient transport and fetal *β*-cell development (11, 12), thereby creating a pro-macrosomic milieu independent of gestational diabetes mellitus (GDM). However, the interaction between pre-pregnancy BMI and glycemic status remains uncertain. Although GDM is a well-established risk factor for macrosomia (13, 14), emerging evidence suggests that a substantial proportion of cases occur in non-GDM pregnancies, indicating etiological heterogeneity that warrants further investigation (15, 16). Current clinical guidelines and research employ uniform BMI-based management strategies to reduce adverse pregnancy outcomes (17, 18), potentially overlooking effect modification by GDM status. This “one-size-fits-all” approach raises critical questions about whether GDM status modifies the pre-pregnancy BMI-macrosomia relationship. Advances in precision medicine have introduced new opportunities for risk-stratified pregnancy management, as exemplified by predictive models such as the GDM-specific risk score developed by Cooray et al. (19) to forecast adverse outcomes. Nevertheless, insufficient evidence exists to justify distinct management approaches for GDM and non-GDM populations regarding pre-pregnancy BMI-related macrosomia prevention. Robust comparisons of BMI-associated risks across glycemic strata are urgently needed to inform precision-based guidelines.

This study aims to delineate the associations between pre-pregnancy BMI and macrosomia in GDM and non-GDM populations using a prospectively collected first-trimester pregnancy cohort. By stratifying analyses according to glycemic status, we seek to determine whether the magnitude and direction of pre-pregnancy BMI-macrosomia relationships differ between these groups, particularly across demographic subgroups. This research may advocate for distinct pre-pregnancy BMI-based management thresholds in GDM vs. non-GDM pregnancies, advancing the paradigm of precision perinatal care. Given the escalating global burdens of obesity and GDM, addressing these critical knowledge gaps holds immediate relevance for maternal-child health policy and practice.

## 2 Materials and methods

### 2.1 Study design and participant enrollment

**Figure 1:**
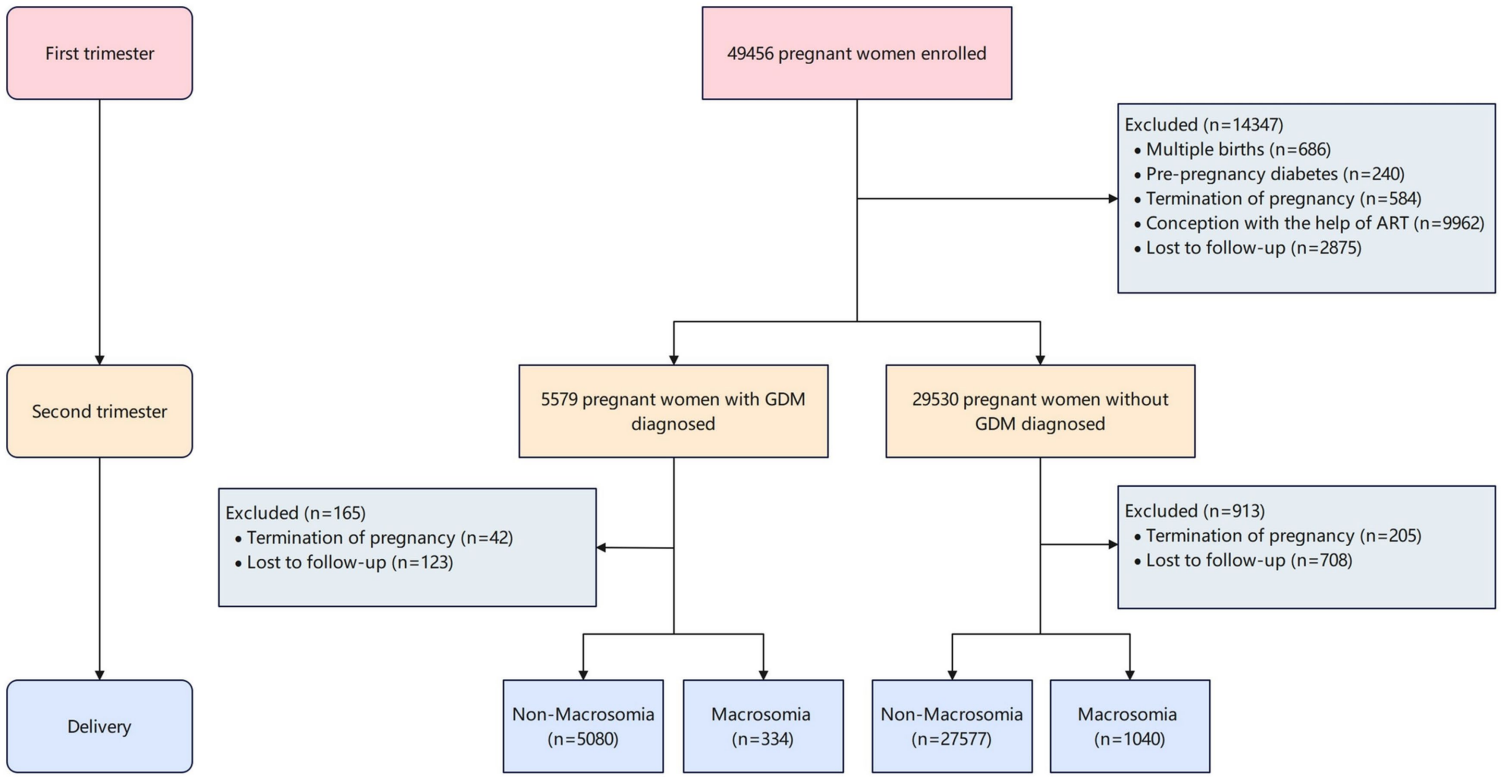
Flow chart showing study design and inclusion of participants.

This prospective cohort study was conducted at Hunan Provincial Maternal and Child Health Care Hospital in central China between March 13, 2013, and December 31, 2019. Pregnant women aged ≥ 18 years who initiated prenatal care before 14 weeks of gestation and planned to receive ongoing care and deliver at the study hospital were enrolled. Participants were recruited from outpatient departments (reproductive medicine, obstetrics, and ultrasonography) following written informed consent. The study protocol was approved by the Ethics Committee of Xiangya School of Public Health, Central South University (Approval No. XYGW-2018-36). Women who conceived through assisted reproductive technologies (ART) were excluded to minimize confounding. At 24–28 weeks of gestation, participants underwent a 75 g oral glucose tolerance test (OGTT) following International Association of Diabetes and Pregnancy Study Groups (IADPSG) criteria. Plasma glucose levels (fasting, 1-h, and 2-h post-load) were measured using an automated analyzer (Toshiba TBA-120FR, Tokyo, Japan). GDM was diagnosed if any value met the thresholds: fasting ≥ 5.1 mmol/L, 1-h ≥ 10.0 mmol/L, or 2-h ≥ 8.5 mmol/L. After applying exclusion criteria (Figure 1), the final analytical cohort consisted of 34,031 women.

### 2.2 Data collection

A structured questionnaire was administered by trained interviewers through face-to-face interviews and telephone follow-ups to collect sociodemographic characteristics, behavioral habits, and lifestyle factors during early pregnancy (<14 weeks). The questionnaire, which has been previously published (20), included variables such as maternal age, residence (urban/rural), ethnicity (Han Chinese/minority groups), parity, gravidity, height and weight values before conception, education level, monthly household income per capita, smoking status, alcohol intake, and folic acid supplementation. Self-reported data (including maternal age, residence, ethnicity, parity, gravidity, education level, height and weight values before conception, and folic acid supplementation) were cross-checked with electronic medical records (EMRs) for accuracy. Pregnancy complications, clinical measurements, and delivery outcomes were systematically extracted from the hospital’s EMR system. Quality control measures included duplicate data entry and periodic audits conducted by an independent team to resolve discrepancies.

### 2.3 Exposures and covariates

Pre-pregnancy BMI (weight[kg]/height[m]^2^) served as the primary exposure, calculated from self-reported values of weight and height before conception by participants at the first prenatal visit. Maternal pre-pregnancy BMI was categorized according to World Health Organization (WHO) criteria (21): underweight (<18.5 kg/m^2^), normal (18.5–24.9 kg/m^2^), overweight (25.0–29.9 kg/m^2^), and obese (≥30.0 kg/m^2^). Covariates included maternal age (calculated from national identification card birthdates), ethnicity, education level (junior high school or below, high school/technical secondary school, college degree, bachelor’s degree or above), residence, gravidity (1 vs. ≥2 times), parity (primiparous/multiparous), monthly household income per capita (≤CNY 2,500, CNY 2,501 to 5,000, >CNY 5,000), smoking status (active [self-reported tobacco use] or passive [≥1 day/week secondhand smoke exposure]) (22), alcohol intake (any consumption during early pregnancy) (22), folic acid supplementation (self-reported whether folic acid was used before or during pregnancy) (22), gestational weight gain, gestational age at delivery, and infant sex (male/female).

### 2.4 Outcomes

The primary outcome was macrosomia, defined as birth weight exceeding 4,000 g (23). Birth weight and neonatal outcomes were extracted from EMRs and cross-checked by obstetricians blinded to exposure status.

### 2.5 Statistical analysis

Data management utilized EpiData 3.1 with double-entry verification. Participants were stratified into two independent groups based on GDM status. Continuous variables with normal distributions are expressed as mean (SD); categorical variables as counts (percentages). Pre-pregnancy BMI categories comparisons employed analysis of variance test for continuous variables and χ^2^/Fisher’s exact tests for categorical variables. The Mantel–Haenszel χ^2^ test was used to evaluate trends for macrosomia incidence by different pre-pregnancy BMI categories. Multivariable logistic regression models were developed to evaluate associations between pre-pregnancy BMI (per 1-unit increase and categories) and macrosomia, stratified by GDM status, using odds ratios (ORs) with 95% confidence intervals. Moreover, the restricted cubic spline (RCS) regression model with assumed three knots was used to address the potential nonlinearity of the association between pre-pregnancy BMI and macrosomia. Further stratified analyses according to age (<35 year and ≥35 years), gravidity (1/≥2 times), parity (primiparous/multiparous), and infant sex (male/female) in the subsequent pregnancy were conducted to identify the consistency of the impact of pre-pregnancy BMI for macrosomia among GDM and non-GDM pregnancies. The interaction between pre-pregnancy BMI and stratified variables was further tested.

## 3 Results

**Figure 2:**
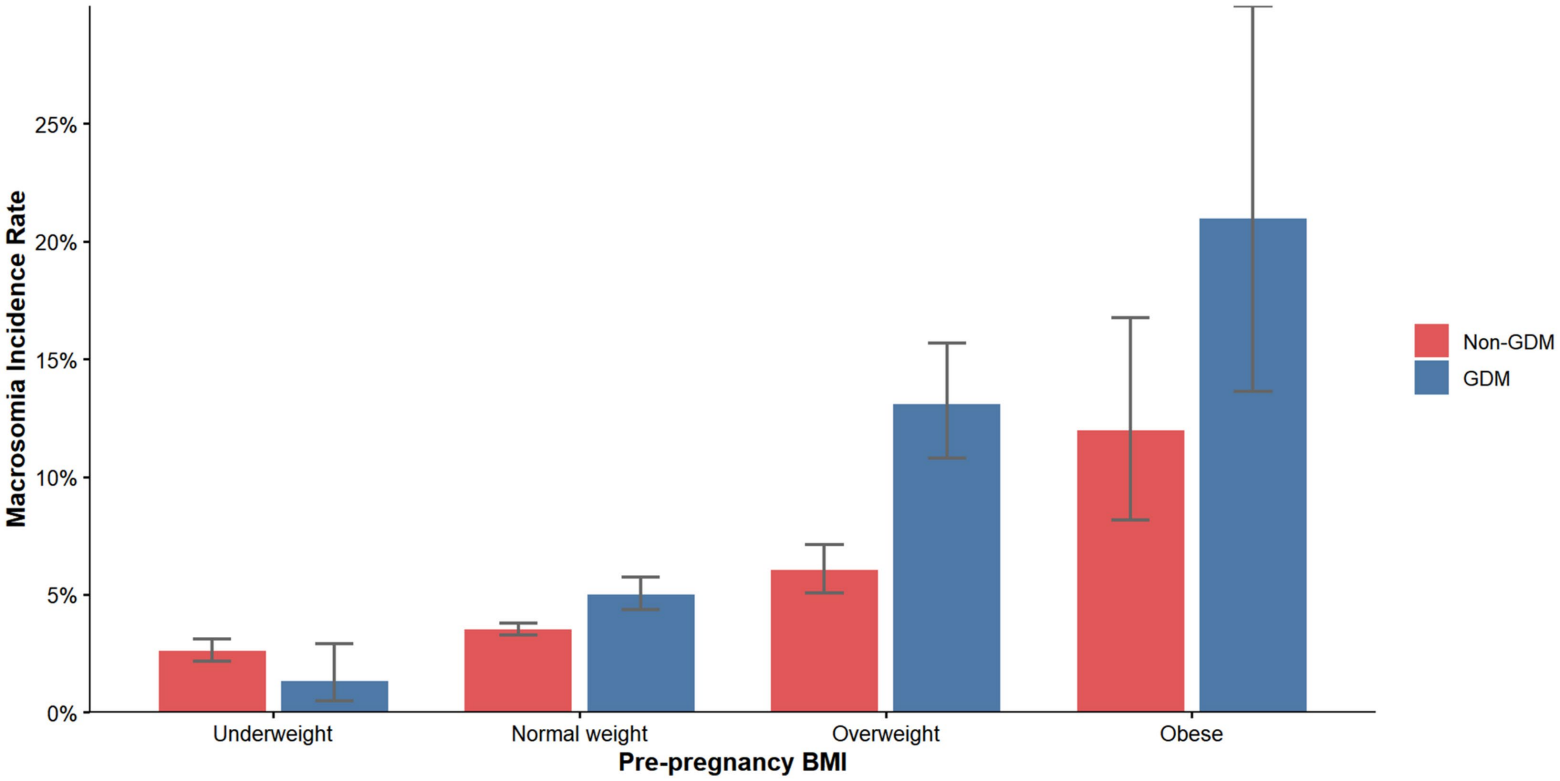
Incidence of macrosomia according to pre-pregnancy BMI categories. BMI, body mass index; GDM, gestational diabetes mellitus.

A total of 34,031 participants were included in this first-trimester pregnancy cohort, with a mean maternal age of 32.43 ± 4.52 years and a mean pre-pregnancy BMI of 22.20 ± 3.16 kg/m^2^. Among these, 5,414 women (15.9%) were diagnosed with gestational diabetes mellitus (GDM), while 28,617 (84.1%) constituted the non-GDM group. Macrosomia was identified in 1,374 neonates (4.0%), with significantly higher incidence rates observed in GDM pregnancies (334 cases, 6.2%) compared to non-GDM pregnancies (1,040 cases, 3.6%). The incidence of macrosomia demonstrated a marked gradient across pre-pregnancy BMI categories (Figure 2), ranging from 1.34% (GDM) and 2.60% (non-GDM) in underweight women to 21.0% (GDM) and 12.0% (non-GDM) in obese women (*P* for trend < 0.001).

### 3.1 Baseline characteristics stratified by pre-pregnancy BMI and GDM status

Baseline characteristics of GDM and non-GDM participants, stratified by pre-pregnancy BMI categories, are presented in Table 1. Among women with GDM, significant differences were observed across BMI categories for maternal age (*p* < 0.001), advanced maternal age (*p* < 0.001), gravidity (*p* < 0.001), parity (*p* < 0.001), ethnicity (*p* < 0.001), education level (*p* = 0.002), and gestational weight gain (*p* < 0.001). In non-GDM pregnancies, differences were noted in maternal age (*p* < 0.001), advanced maternal age (*p* < 0.001), gravidity (*p* < 0.001), parity (*p* < 0.001), education level (*p* < 0.001), gestational weight gain (*p* < 0.001), smoking status (*p* = 0.042), infant sex (*p* < 0.001), and gestational age at delivery (*p* = 0.006). A dose–response relationship between higher pre-pregnancy BMI and increased birth weight was observed in both groups (*p* < 0.001).

**Table 1:** Baseline characteristics of GDM and non-GDM patients grouped according to pre-pregnancy BMI categories.

Characteristics	GDM						Non-GDM					
	Overall (*N* = 5,414)	Underweight (*N* = 448)	Normal weight (*N* = 4,090)	Overweight (*N* = 771)	Obesity (*N* = 105)	*p*-value	Overall (*N* = 28,617)	Underweight (*N* = 4,459)	Normal weight (*N* = 21,764)	Overweight (*N* = 2,152)	Obesity (*N* = 242)	*p*-value
Age at delivery (years), mean (SD)	32.43 (4.52)	30.24 (3.97)	32.63 (4.49)	32.77 (4.50)	31.74 (5.41)	< 0.001	30.87 (4.47)	29.23 (4.09)	31.11 (4.45)	31.62 (4.73)	32.63 (3.88)	< 0.001
Advanced maternal age (≥ 35 years), *n* (%)						< 0.001						< 0.001
No	3,695 (68.2)	385 (85.9)	2,720 (66.5)	512 (66.4)	78 (74.3)		22,687 (79.3)	3,980 (89.3)	16,979 (78.0)	1,568 (72.9)	160 (66.1)	
Yes	1719 (31.8)	63 (14.1)	1,370 (33.5)	259 (33.6)	27 (25.7)		5,930 (20.7)	479 (10.7)	4,785 (22.0)	584 (27.1)	82 (33.9)	
Residence, *n* (%)						0.270						0.295
Urban	3,429 (63.3)	299 (66.7)	2,592 (63.4)	471 (61.1)	67 (63.8)		17,598 (61.5)	2,768 (62.1)	13,339 (61.3)	1,330 (61.8)	161 (66.5)	
Rural	1985 (36.7)	149 (33.3)	1,498 (36.6)	300 (38.9)	38 (36.2)		11,019 (38.5)	1,691 (37.9)	8,425 (38.7)	822 (38.2)	81 (33.5)	
Pre-pregnancy BMI (kg/m^2^), mean (SD)	22.20 (3.16)	17.45 (0.92)	21.59 (1.72)	26.80 (1.32)	32.73 (2.21)	< 0.001	21.15 (2.85)	17.48 (0.89)	21.22 (1.66)	26.73 (1.25)	32.42 (5.45)	< 0.001
Gravidity (times), *n* (%)						< 0.001						< 0.001
1	1,477 (27.3)	191 (42.6)	1,056 (25.8)	196 (25.4)	34 (32.4)		9,000 (31.4)	1795 (40.3)	6,674 (30.7)	493 (22.9)	38 (15.7)	
≥2	3,937 (72.7)	257 (57.4)	3,034 (74.2)	575 (74.6)	71 (67.6)		19,617 (68.6)	2,664 (59.7)	15,090 (69.3)	1,659 (77.1)	204 (84.3)	
Parity, *n* (%)						< 0.001						< 0.001
Primiparous	2,481 (45.8)	316 (70.5)	1773 (43.3)	342 (44.4)	50 (47.6)		13,926 (48.7)	2,660 (59.7)	10,357 (47.6)	817 (38.0)	92 (38.0)	
Multiparous	2,933 (54.2)	132 (29.5)	2,317 (56.7)	429 (55.6)	55 (52.4)		14,691 (51.3)	1799 (40.3)	11,407 (52.4)	1,335 (62.0)	150 (62.0)	
Ethnicity, *n* (%)						< 0.001						0.391
Han	5,339 (98.6)	448 (100)	4,032 (98.6)	765 (99.2)	94 (89.5)		28,244 (98.7)	4,410 (98.9)	21,471 (98.7)	2,126 (98.8)	237 (97.9)	
Minority	75 (1.4)	0 (0)	58 (1.4)	6 (0.8)	11 (10.5)		373 (1.3)	49 (1.1)	293 (1.3)	26 (1.2)	5 (2.1)	
Education level, *n* (%)						0.002						< 0.001
Junior high and below	417 (7.7)	21 (4.7)	318 (7.8)	63 (8.2)	15 (14.3)		2,116 (7.4)	346 (7.8)	1,475 (6.8)	242 (11.2)	53 (21.9)	
High school or technical secondary school	1,448 (26.7)	123 (27.5)	1,065 (26.0)	220 (28.5)	40 (38.1)		8,227 (28.7)	1,215 (27.2)	6,133 (28.2)	785 (36.5)	94 (38.8)	
College degree	2,597 (48)	216 (48.2)	1985 (48.5)	362 (47)	34 (32.4)		12,963 (45.3)	2,170 (48.7)	9,903 (45.5)	827 (38.4)	63 (26)	
Bachelor’s degree or above	952 (17.6)	88 (19.6)	722 (17.7)	126 (16.3)	16 (15.2)		5,311 (18.6)	728 (16.3)	4,253 (19.5)	298 (13.8)	32 (13.2)	
Per-caput monthly family income (CNY), *n* (%)						0.541						0.416
≤2,500	944 (17.4)	75 (16.7)	716 (17.5)	136 (17.6)	17 (16.2)		4,936 (17.2)	748 (16.8)	3,765 (17.3)	383 (17.8)	40 (16.5)	
2,500 to 5,000	2,860 (52.8)	242 (54.0)	2,170 (53.1)	386 (50.1)	62 (59.0)		15,300 (53.5)	2,421 (54.3)	11,603 (53.3)	1,158 (53.8)	118 (48.8)	
>5,000	1,610 (29.7)	131 (29.2)	1,204 (29.4)	249 (32.3)	26 (24.8)		8,381 (29.3)	1,290 (28.9)	6,396 (29.4)	611 (28.4)	84 (34.7)	
Alcohol intake, *n* (%)						0.054						0.091
No	5,323 (98.3)	437 (97.5)	4,016 (98.2)	765 (99.2)	105 (100)		28,210 (98.6)	4,413 (99)	21,438 (98.5)	2,122 (98.6)	237 (97.9)	
Yes	91 (1.7)	11 (2.5)	74 (1.8)	6 (0.8)	0 (0)		407 (1.4)	46 (1)	326 (1.5)	30 (1.4)	5 (2.1)	
Smoking, *n* (%)						0.654						0.042
No	4,991 (92.2)	409 (91.3)	3,770 (92.2)	717 (93.0)	95 (90.5)		26,267 (91.8)	4,050 (90.8)	20,000 (91.9)	1995 (92.7)	222 (91.7)	
Yes	423 (7.8)	39 (8.7)	320 (7.8)	54 (7.0)	10 (9.5)		2,350 (8.2)	409 (9.2)	1764 (8.1)	157 (7.3)	20 (8.3)	
Folic acid supplementation, *n* (%)						0.588						0.333
No	236 (4.4)	21 (4.7)	182 (4.4)	31 (4.0)	2 (1.9)		1,308 (4.6)	191 (4.3)	1,018 (4.7)	92 (4.3)	7 (2.9)	
Yes	5,178 (95.6)	427 (95.3)	3,908 (95.6)	740 (96.0)	103 (98.1)		27,309 (95.4)	4,268 (95.7)	20,746 (95.3)	2060 (95.7)	235 (97.1)	
Infant sex, *n* (%)						0.313						< 0.001
Male	2,732 (50.5)	211 (47.1)	2090 (51.1)	382 (49.5)	49 (46.7)		15,180 (53.0)	2,406 (54.0)	11,407 (52.4)	1,193 (55.4)	174 (71.9)	
Female	2,682 (49.5)	237 (52.9)	2000 (48.9)	389 (50.5)	56 (53.3)		13,437 (47.0)	2053 (46.0)	10,357 (47.6)	959 (44.6)	68 (28.1)	
Gestational age at delivery (weeks), mean (SD)	38.45 (2.14)	38.24 (2.30)	38.45 (2.11)	38.56 (2.15)	38.58 (2.25)	0.078	38.43 (2.12)	38.34 (2.21)	38.45 (2.09)	38.36 (2.18)	38.55 (1.98)	0.006
Gestational weight gain (g), mean (SD)	12.32 (4.69)	13.86 (4.52)	12.49 (4.59)	10.91 (4.76)	9.68 (5.40)	< 0.001	13.81 (4.72)	14.62 (4.69)	13.87 (4.61)	11.81 (5.01)	11.43 (6.23)	< 0.001
Birth weight (g), mean (SD)	3160.32 (594.28)	2974.71 (572.86)	3157.33 (577.48)	3238.60 (661.75)	3493.86 (557.46)	< 0.001	3142.46 (544.24)	3041.11 (517.02)	3159.2 (535.17)	3169.17 (641.18)	3267.64 (674.39)	< 0.001

BMI, body mass index; GDM, gestational diabetes mellitus.

### 3.2 Association between pre-pregnancy BMI and macrosomia

Logistic regression analyses demonstrated a strong positive association between pre-pregnancy BMI and macrosomia in both GDM and non-GDM populations (Table 2). In unadjusted models, each 1-unit increase in BMI was associated with 20% higher odds of macrosomia in GDM (OR: 1.20 [95% CI: 1.16, 1.24], *p* < 0.001) and 10% higher odds in non-GDM (OR 1.10 [1.08, 1.12], *p* < 0.001). After full adjustment for covariates, the associations remained significant and strengthened, particularly in GDM (aOR 1.24 [1.20, 1.28], *p* < 0.001) compared to non-GDM (aOR: 1.13 [1.11, 1.15], *p* < 0.001). Categorical analyses revealed a different pattern. Among GDM pregnancies, pre-pregnancy obesity conferred a 6.80-fold risk of macrosomia (aOR 6.80 [4.02, 11.51], *p* < 0.001) relative to normal pre-pregnancy BMI after full adjustment for covariates, while overweight was associated with a 3.38-fold risk (aOR 3.38 [2.60, 4.39], *p* < 0.001). In non-GDM pregnancies, obesity and overweight were associated with 4.70-fold (aOR 4.70 [3.12, 7.10], *p* < 0.001) and 2.23-fold (aOR 2.23 [1.83, 2.71], *p* < 0.001) increased risks, respectively. Notably, underweight women exhibited significantly reduced risks in both groups (GDM: aOR 0.23 [0.10, 0.53]; non-GDM: aOR 0.65 [0.53, 0.79], *p* < 0.001).

**Table 2:** Odd ratios for macrosomia associated with severity of pre-pregnancy BMI among GDM and non-GDM pregnancies.

Categories	Events (%)	Model 1			Model 2			Model 3		
		OR (95% CI)	*p*-value	*P* for trend	OR (95% CI)	*p*-value	*P* for trend	OR (95% CI)	*p*-value	*P* for trend
GDM										
Continuous variable per 1 unit		1.20 (1.16, 1.24)	< 0.001		1.21 (1.17, 1.25)	< 0.001		1.24 (1.20, 1.28)	< 0.001	
Category	334 (6.2)			< 0.001			< 0.001			< 0.001
Underweight (*N* = 448)	6 (1.3)	0.26 (0.11, 0.58)	0.001		0.27 (0.12, 0.60)	0.002		0.23 (0.10, 0.53)	< 0.001	
Normal weight (*N* = 4,090)	205 (5.0)	Ref.			Ref.			Ref.		
Overweight (*N* = 771)	101 (13.1)	2.86 (2.22, 3.68)	< 0.001		2.91 (2.26, 3.75)	< 0.001		3.38 (2.60, 4.39)	< 0.001	
Obesity (*N* = 105)	22 (21.0)	5.02 (3.08, 8.20)	< 0.001		5.20 (3.14, 8.62)	< 0.001		6.80 (4.02, 11.51)	< 0.001	
Non-GDM										
Continuous variable per 1 unit		1.10 (1.08, 1.12)	< 0.001		1.10 (1.08, 1.12)	< 0.001		1.13 (1.11, 1.15)	< 0.001	
Category	1,040 (3.6)			< 0.001			< 0.001			< 0.001
Underweight (*N* = 4,459)	116 (2.6)	0.73 (0.60, 0.89)	0.002		0.71 (0.58, 0.87)	0.001		0.65 (0.53, 0.79)	< 0.001	
Normal weight (*N* = 21,764)	765 (3.5)	Ref.			Ref.			Ref.		
Overweight (*N* = 2,152)	130 (6.0)	1.77 (1.46, 2.14)	< 0.001		1.82 (1.50, 2.21)	< 0.001		2.23 (1.83, 2.71)	< 0.001	
Obesity (*N* = 242)	29 (12.0)	3.74 (2.52, 5.55)	< 0.001		4.06 (2.72, 6.05)	< 0.001		4.70 (3.12, 7.10)	< 0.001	

OR, odd ratios; GDM, gestational diabetes mellitus. Model 1, unadjusted; Model 2, adjusted for maternal age, residence location, gravidity, parity, per caput monthly family income, ethnicity, and education level; Model 3, adjusted for maternal age, residence location, gravidity, parity, per caput monthly family income, ethnicity, education level, smoking, alcohol intake, folic acid supplementation, gestational weight gain, delivery gestational age in the subsequent pregnancy, and infant sex.

**Figure 3:**
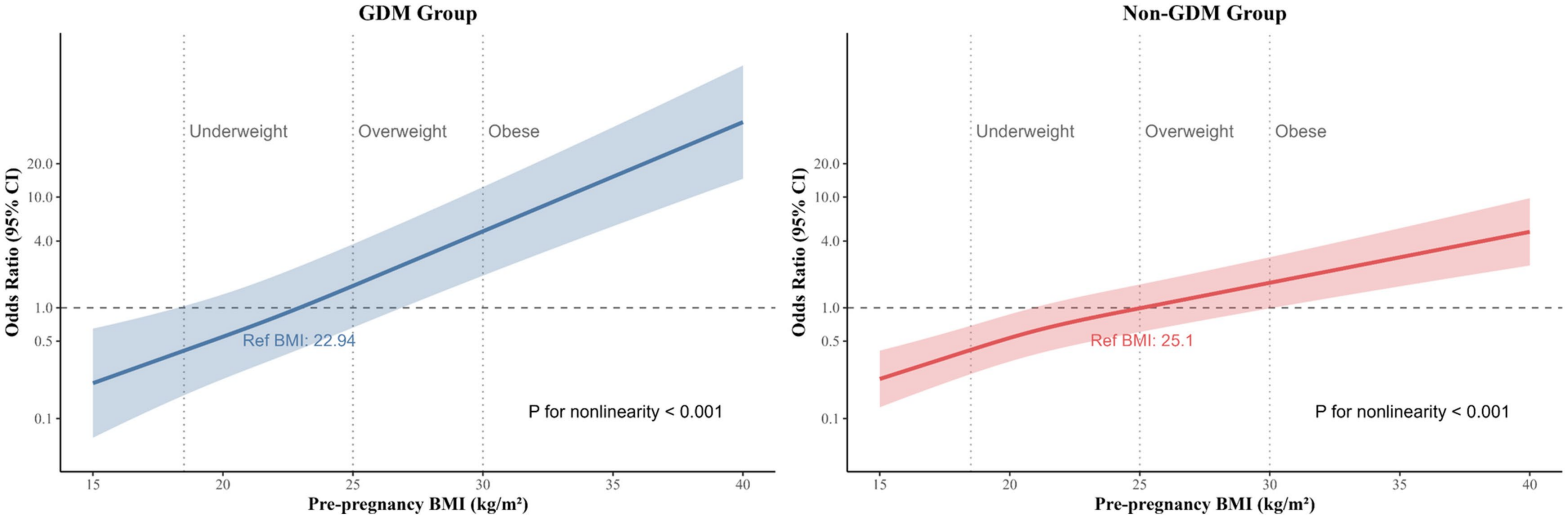
Restricted cubic spline of the association between pre-pregnancy BMI and macrosomia among GDM and non-GDM pregnancies. Heavy central lines represent the estimated adjusted odd ratios, with shaded ribbons denoting 95% confidence intervals. Pre-pregnancy BMI 22.94 and 25.10 were selected as the reference level for GDM and non-GDM pregnancies, respectively. Pre-pregnancy BMI 18.5, 25.0, and 30.0 was categorized based on the World Health Organization criteria represented by the vertical dotted lines. The horizontal dotted lines represent the odd ratio of 1.0. The model was adjusted for maternal age, residence location, gravidity, parity, per caput monthly family income, ethnicity, education level, smoking, alcohol intake, folic acid supplementation, gestational weight gain, delivery gestational age in the subsequent pregnancy, and infant sex. BMI, body mass index; CI, confidence interval; GDM, gestational diabetes mellitus, OR, odd ratios.

RCS models further elucidated the nonlinear associations (Figure 3). In GDM pregnancies, macrosomia risk increased sharply beyond a pre-pregnancy BMI threshold of 25.0 kg/m^2^, with near-exponential escalation at BMI > 30.0 kg/m^2^. In non-GDM pregnancies, the risk curve was less pronounced but remained significant (*P* for nonlinearity < 0.001), indicating that even moderate pre-pregnancy BMI elevations (25.0–29.9 kg/m^2^) in pregnant women with normal glucose metabolism meaningfully contribute to fetal overgrowth.

### 3.3 Subgroup and interaction analyses

**Figure 4:**
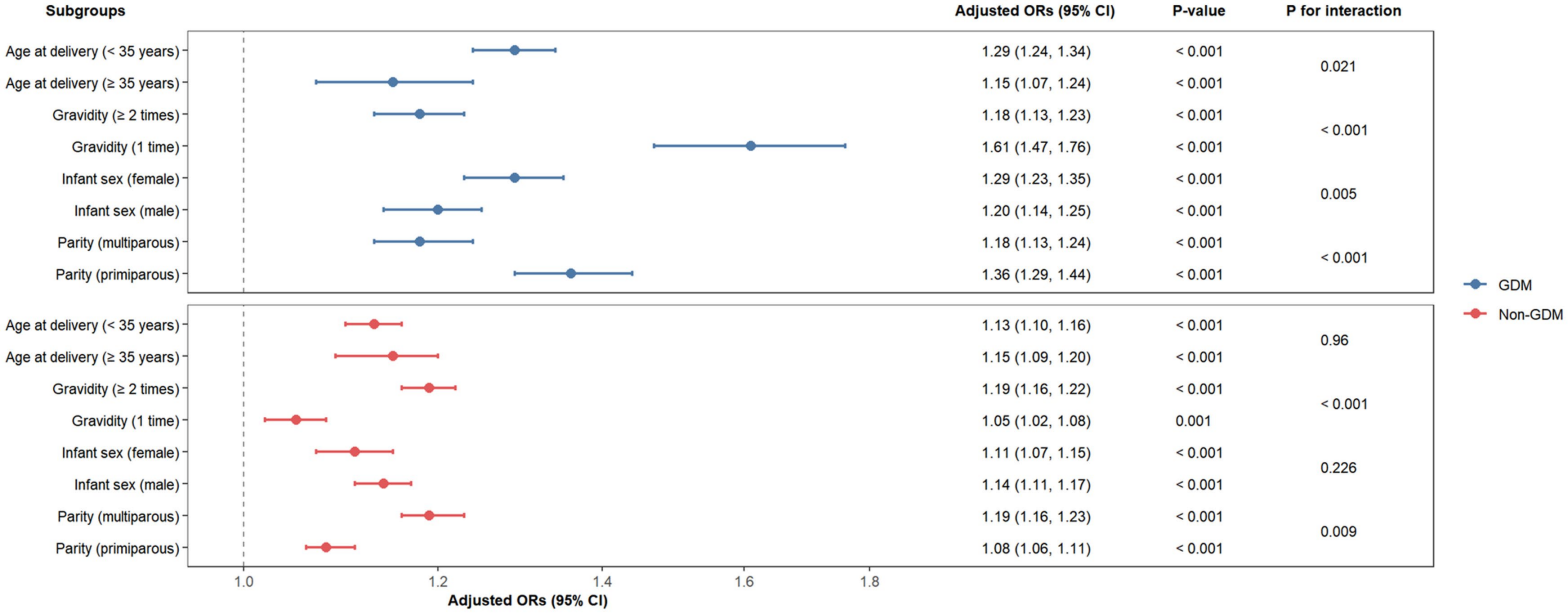
Forest plots of odd ratios for macrosomia in different subgroups among GDM and non-GDM pregnancies. CI, confidence interval; GDM, gestational diabetes mellitus; OR, odd ratios.

We further conducted a stratified analyses of the relationship between pre-pregnancy BMI and a macrosomia according to the potential modifiers, including maternal age, gravidity, parity, and infant sex (Figure 4). Pre-pregnancy BMI was significantly associated with increased macrosomia risk across all subgroups in both GDM and non-GDM populations (*p* < 0.05). Significant interactions were observed in GDM pregnancies, and the association was stronger in women < 35 years (OR per 1-unit BMI increase: 1.29 vs. ≥ 35 years: 1.15; *P* for interaction = 0.021), primigravida (OR: 1.61 vs. multigravida: 1.18; *P* for interaction < 0.001), primiparous (OR: 1.36 vs. multiparous: 1.18; *P* for interaction < 0.001), and female infants (OR: 1.29 vs. male infants: 1.20; *P* for interaction = 0.005). In non-GDM pregnancies, parity (multiparous: OR: 1.19 vs. primiparous: 1.08; *P* for interaction < 0.001) and gravidity (≥ 2 times: OR: 1.19 vs. 1 time: 1.05; *P* for interaction = 0.009) modified the pre-pregnancy BMI-macrosomia relationship. No significant interactions were detected for maternal age or infant sex in non-GDM pregnancies (*P* for interaction > 0.05). These findings highlight context-specific risk profiles, emphasizing the need for tailored interventions in high-risk subgroups.

## 4 Discussion

This prospective cohort study delineates critical differences in the association between pre-pregnancy BMI and macrosomia risk among GDM and non-GDM populations. We observed a pronounced dose–response relationship between elevated pre-pregnancy BMI and macrosomia in both groups. Compared to women with normal pre-pregnancy BMI, those classified as overweight (aOR: 3.38 for GDM; 2.23 for non-GDM) or obese (aOR: 6.80 for GDM; 4.70 for non-GDM) exhibited significantly increased risks of giving birth to a macrosomia. A protective effect of lower pre-pregnancy BMI against macrosomia was also identified. RCS models further revealed nonlinear risk trajectories, with distinct patterns between GDM and non-GDM pregnancies. These findings highlight the need for glycemic status-specific pre-pregnancy BMI thresholds in macrosomia prevention strategies.

The differential risk gradients observed between GDM and non-GDM populations call into question the current “one-size-fits-all” approach to BMI-based prenatal care. Although it is well known that pre-pregnancy overweight/obesity universally predisposes to fetal overgrowth (24–26), the magnitude of risk escalation in GDM pregnancies suggests that hyperglycemia and insulin resistance exacerbate adiposity-driven pathways. This aligns with mechanistic studies implicating adipose-derived cytokines (e.g., leptin, TNF-*α*) in placental nutrient dysregulation and fetal *β*-cell hyperplasia, processes intensified by GDM (27–29). Contrary to prior research attributing the BMI-macrosomia association primarily to hyperglycemic features (30), our findings clarify that elevated pre-pregnancy BMI independently drives fetal overgrowth in non-GDM pregnancies, potentially through mechanisms such as subclinical insulin resistance or inflammatory pathways (31). The nonlinear associations identified by RCS models refine clinical insights: inflection points in GDM pregnancies imply that WHO BMI categories, designed for general populations, may inadequately capture risk thresholds in metabolically high-risk subgroups. This advocates for reevaluating pre-pregnancy BMI cutoffs in GDM-specific guidelines (32), potentially integrating continuous BMI metrics with glycemic parameters for risk prediction. Furthermore, persistent risks in non-GDM women with overweight/obesity highlight the public health priority of universal preconception weight optimization. Strikingly, underweight women exhibited a 77% risk reduction in GDM pregnancies vs. 35% in non-GDM pregnancies. Although this negative association, also observed in previous studies (8, 9), aligns with reduced nutrient availability limiting fetal growth, it may mask risks of intrauterine growth restriction or maternal undernutrition—factors not captured in this study. It still needs to be interpreted with caution.

Subgroup analyses not only validated the robustness of the results but also identified key factors for redefining risk stratification. These *post hoc* observations revealed intriguing patterns. In GDM pregnancies, younger women (<35 years), primigravida, primiparous individuals, and those carrying female infants exhibited elevated macrosomia risks per unit BMI increase. In the context of a high-risk state of metabolic disorders, primiparous women and first-time pregnancies may have a synergistic effect with higher pre pregnancy BMI, exacerbating fetal overgrowth. Conversely, in non-GDM pregnancies, multiparity and multigravidity amplified pre pregnancy BMI-associated risks, likely reflecting cumulative metabolic stressors from prior pregnancies or sociodemographic factors influencing weight retention (33–35). These findings challenge the assumption of uniform pathophysiology across glycemic strata. The stronger association between pre-pregnancy BMI and macrosomia in younger GDM women suggests age-related metabolic vulnerabilities, further investigations are needed to elucidate the exact mechanisms underlying this phenomenon. Older women, despite higher baseline metabolic risk, might benefit from age-related changes in placental efficiency (36, 37). Although the susceptibility of female fetal under the background of hyperglycemia was also observed in a previous pregnant women cohort (38, 39), the present findings lack direct mechanistic measurements (e.g., placental epigenetics, fetal insulin levels). It is worth noting that these observational associations should be viewed as hypothesis-generating, highlighting priority areas for future mechanistic research rather than confirming causal pathways.

While this study provides novel insights into the interaction between pre-pregnancy BMI, GDM status, and macrosomia risk across demographic subgroups, several limitations warrant acknowledgment. First, although our interaction-focused analysis of demographic modifiers in GDM and non-GDM cohorts represents a distinct methodological advance, we acknowledge that prior research has examined related mediation pathways (e.g., hyperglycemia as mediator between pre-pregnancy BMI and macrosomia) and effect modification in general populations (8, 30). Second, despite adjusting for key sociodemographic, behavioral, and clinical confounders, residual confounding from unmeasured lifestyle factors, including detailed dietary patterns, physical activity levels, gestational weight gain trajectories, and psychosocial aspects, may persist. Such factors could partially account for subgroup disparities and potentially overestimate or underestimate BMI-associated risk estimates. Third, pre-pregnancy weight and height data were self-reported, introducing possible information bias. Although a previous study has demonstrated reasonable agreement between self-reported and anthropometric data, systematic underestimation of weight (particularly in pregnant women) remains a well-documented limitation (40). Future multiethnic cohorts should incorporate objectively measured pre-pregnancy anthropometrics, longitudinal lifestyle monitoring, and biomarker validation to address these constraints. Additionally, intervention trials are needed to test whether stratified pre-pregnancy BMI management in GDM pregnancies reduces macrosomia incidence more effectively than uniform approaches.

## 5 Conclusion

This study provides compelling evidence supporting the implementation of glycemic status-specific pre-pregnancy BMI thresholds for macrosomia prevention. By integrating these insights into clinical practice, preconception and antenatal care can be more effectively individualized by healthcare providers, thereby mitigating the escalating dual burdens of obesity and GDM on maternal and child health. As precision medicine continues to transform perinatal care, our findings highlight the imperative to advance beyond population-wide guidelines by adopting risk-stratified strategies tailored to address the unique pathophysiological mechanisms of high-risk subgroups.

## Data Availability

The raw data supporting the conclusions of this article will be made available by the authors, without undue reservation.
